# Evaluation of Deflection Prediction Models for RC Beams with High-Strength Steel Reinforcement

**DOI:** 10.3390/ma19081576

**Published:** 2026-04-14

**Authors:** Gintaris Kaklauskas, Aqib Ahmed, Adas Meskenas, Juozas Valivonis, Aleksandr Sokolov

**Affiliations:** 1 Department of Reinforced Concrete Structures and Geotechnical Engineering, Vilnius Tech—Vilnius Gediminas Technical University, Saulėtekio al. 11, LT-10223 Vilnius, Lithuania; aqib.ahmed@vilniustech.lt (A.A.); adas.meskenas@vilniustech.lt (A.M.); juozas.valivonis@vilniustech.lt (J.V.); 2 Laboratory of Innovative Building Structures, Vilnius Tech—Vilnius Gediminas Technical University, Saulėtekio al. 11, LT-10223 Vilnius, Lithuania; aleksandr.sokolov@vilniustech.lt

**Keywords:** reinforced concrete, high-strength steel, deflection, validation, serviceability, curvature analysis, tension stiffening, statistics

## Abstract

**Highlights:**

- RC beams with HSS bars have lower tension stiffening compared to standard steel bars.
- On average, the design codes tend to underestimate curvature in RC beams with HSS bars.
- The predicted mean normalized curvature (*κ_th_*/*κ_exp_*) ranged from 0.99 to 0.81 for different models.
- The coefficient of variation ranged between 17.8% and 24.9% for different models.

**Abstract:**

The modern construction industry has witnessed a marked shift towards the utilization of high-strength steel reinforcement, exhibiting yield strengths exceeding 600 MPa in reinforced concrete structures. Tension stiffening is a critical factor for accurate prediction of deflection and crack width. The current study evaluates the accuracy of state-of-the-art models in predicting curvature in Reinforced Concrete (RC) beams reinforced with high-strength steel (HSS) bars. This study employed three design code methods (Eurocode 2, ACI 318-14, and ACI 318-19) and two other models: the Bischoff model and Kaklauskas and Sokolov’s model. An RC beam with HSS bars was tested, and experimental data on another 63 RC beams reinforced with HSS rebars were collected from various published studies. The test data ranged in various geometrical and material characteristics and were evaluated across a wide range of steel stress intervals. An inverse analysis was carried out to calculate the resultant internal force of tensile concrete (tension stiffening) from the experimental moment–curvature diagram. The inverse analysis demonstrated that the fully cracked RC section reached stiffness at a bending moment of about 3*M_cr_*, where *M_cr_* is the cracking bending moment predicted according to the EC2 design code. Statistical analysis showed that the predicted mean normalized curvature (*κ_th_*/*κ_exp_*) across several reinforcement stress levels ranged from 0.99 to 0.81 for different models. The design codes tend to underestimate curvature. The coefficients of variation ranged between 17.8% and 24.9% for different models.

## 1. Introduction

In reinforced concrete structures, serviceability criteria (deflection and crack control) often govern design rather than ultimate strength criteria. Recently, high-strength steel with a yield strength exceeding 500 MPa has seen widespread adoption due to reduced reinforcement area, decreased steel congestion, and improved structural efficiency [1,2,3]. In the modern construction industry, HSS grades with *f_y_* = 600–830 MPa are increasingly employed compared to conventional steel with *f_y_* = 400–500 MPa for applications validated under static conditions [2,4,5,6]. However, HSS exhibits fundamentally different mechanical behavior compared to conventional steel, including a reduction in ductility ratio and altered stress–strain characteristics [3,4,7]. Due to these material differences, the bond-slip mechanism, crack spacing, and tension stiffening are affected, which directly influences the serviceability behavior [1,2,8]. Experimental investigations demonstrate that HSS RC beams at service loads show larger deflections and wider cracks than those predicted by current design codes [1,2,4,5], indicating systematic underprediction despite an adequate ultimate strength capacity.

Current design code provisions [9,10,11] consider simple formulations of the effective moment of inertia or curvature that interpolate between the properties of uncracked and fully cracked sections. The most widely accepted formula of the effective moment of inertia proposed by Branson [12] takes the following form:

(1) $$I_{e}={\left(\frac{M_{cr}}{M}\right)}^{3}I_{g}+\left[1-{\left(\frac{M_{cr}}{M}\right)}^{3}\right]I_{cr}\leq I_{g}$$

where *M_cr_*, *M*, *I_g_*, and *I_cr_* are the cracking moment, applied moment, gross moment of inertia, and fully cracked moment of inertia, respectively.

Bischoff [8] reformulated this approach with improved accuracy for lightly reinforced members, refining it by establishing the relationship of the effective moment of the inertia to moment ratio:

(2) $$I_{e}=\frac{I_{cr}}{1-{\left(\frac{M_{cr}}{M}\right)}^{2}\left[1-\frac{I_{cr}}{I_{g}}\right]}\leq I_{g}$$

The above relationship was adopted in ACI 318-19, assuming a multiplier of 2/3 for the *M_cr_*/*M* ratio. To predict curvature, Eurocode 2 uses the interpolation formulae relating to the uncracked and fully cracked states:

(3) $$\kappa =\left(1-\zeta \right)\kappa_{1}+\zeta \kappa_{2}$$

(4) $$\zeta =1-\beta_{1}\beta_{2}{\left(\frac{M_{cr}}{M}\right)}^{2}$$

where *κ*_1_ and *κ*_2_ are the uncracked and fully cracked instantaneous curvatures, respectively; ζ is the coefficient of distribution between uncracked and fully cracked states ranging between 0 (uncracked) and 1 (fully cracked); *β*_1_ is the coefficient for considering the bonding properties of reinforcement (when the steel bar is ribbed, *β*_1_ = 1.0); and *β*_2_ is the coefficient considering the type of load (taking 1 for short-term load).

It should be noted that the design code approaches were calibrated for conventional steel and an empirically derived correction factor-based formulation, which may not capture the tension stiffening behaviors of HSS reinforcement [3,8].

Despite HSS’s advantages, current design codes and methods provide limited guidance for *f_y_* > 600 MPa, relying on formulations developed for conventional steel (*f_y_* = 400–430 MPa) [3]. Sha et al. [4] performed a test on 17 full-scale beam specimens with HRB 635 steel (*f_y_* = 635 MPa) and demonstrated that both GB 50010-2010 and ACI 318-19 systematically underestimate deflections of HSS RC beams. Experimental studies confirmed that HSS exhibits fundamentally different deformation behavior due to altered stress–strain characteristics and reduced ductility [4,7], so an accurate deflection prediction method still needs to be validated for *f_y_* > 600 MPa [13]. Due to this systematic underprediction and reduced ductility of HSS members, a mechanistically sound deflection prediction method must be specifically validated for HSS reinforcement [4,8,13].

These modern approaches, which involve the direct extraction of tension stiffening relationships from experimental moment curvature response through inverse analysis, were further adopted and refined by researchers.

The accurate prediction of deflection in reinforced concrete flexural members requires an understanding of tension stiffening effects: specifically, the concrete between cracks continues to carry tensile stresses due to bond with reinforcement [14,15,16,17]. Various methodologies have been proposed and employed to quantify tension stiffening effects in reinforced concrete members. Reinforcement-based stress–strain models presented in [18] assume that tensile reinforcement carries additional stresses due to tension stiffening; an inverse technique is introduced in [13,18,19,20,21,22]. More recently, these approaches have been extended to fiber-reinforced concrete members [20]. The inverse analysis is also employed in this study and will be discussed in later sections.

**Figure 1:**
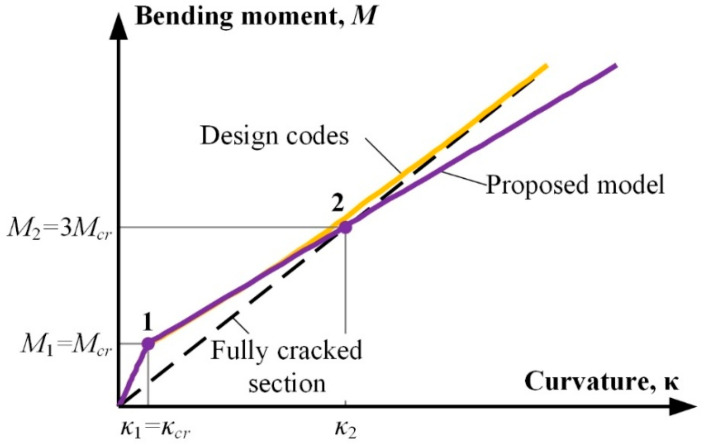
Curvature model by Kaklauskas and Sokolov [13].

In 2021, Kaklauskas and Sokolov [13] calculated the resultant tension stiffening force (*N_ts_*) through an inverse analysis of the moment–curvature response from 69 RC beams and demonstrated that *N_ts_* = 0 (fully cracked state) occurs at *M* = *M*_2_ = 3*M_cr_* independent of the reinforcement ratio, section depth, and concrete strength. Based on this finding, Kaklauskas and Sokolov [13] proposed a simple curvature model, shown in Figure 1 and expressed via Equation (5) as a linear interpolation between two curvature points representing cracking bending moment *M*_1_ = *M_cr_* and bending moment *M*_2_ = 3*M_cr_*. The model has shown better accuracy (mean *κ_pred_*/*κ_exp_* = 0.98 and COV = 0.14) compared to design code methods.

(5) $$\kappa =\kappa +0.5\left(\kappa_{2}-\kappa_{1}\right)\left(\frac{M}{M_{1}}-1\right)$$

where

(6) $$\kappa_{1}=\left(\frac{M_{cr}}{E_{c}I_{g}}\right)\;and\;\kappa_{2}=\left(\frac{{3M}_{cr}}{E_{c}I_{cr}}\right)$$

*κ*_1_ is the curvature calculated at bending moment *M*_1_; *κ*_2_ is the curvature calculated at bending moment *M*_2_.

However, their experimental database was predominantly limited to conventional steel reinforcement with *f_y_* ≤ 500 MPa, with only a small number of beams having *f_y_* > 500 MPa (maximum 632 MPa) [13]. So, the applicability of the peculiar value *β*_0_ = *M*/*M_cr_* to HSS (*f_y_* > 600 MPa) reinforcement is still unexplored [13]. The discovery of the peculiar value *β*_0_ = *M*/*M_cr_* in [13] represents a significant advancement in deflection prediction for conventional steel. However, given that HSS RC beams exhibit systematically larger deflections than those predicted by current design codes [2,4,5], a critical question arises: does the peculiar value *β*_0_ = 3 remain constant for high-strength steel reinforcement (*f_y_* > 600 MPa) or does it vary due to different material characteristics at a higher stress level?

In this study, data on 63 beams are collected from the published literature, and one specimen is experimentally tested. The inverse technique [13,18,19,22] is employed for the evaluation of *β*_0_ = *M*_2_*/M*_1_. The predictive accuracy of the Kaklauskas and Sokolov model [13] for the reinforced concrete bending specimens with HSS (*f_y_* > 600 MPa) is evaluated and compared against the methods presented in [8,9,10,23]. We quantify the statistical performance of each model through mean bias, coefficient of variation, and error distribution analysis. We also identify the conditions under which the approach in [13] provides superior accuracy compared to the conventional moment-based interpolation approach.

## 2. Experimental Program

A full-scale reinforced concrete beam incorporating high-strength longitudinal reinforcement was tested under flexural loading. In parallel, small specimens were examined to determine the material properties, including the compressive and tensile strengths of both concrete and reinforcing steel.

### 2.1. Beam Configuration

**Figure 2:**
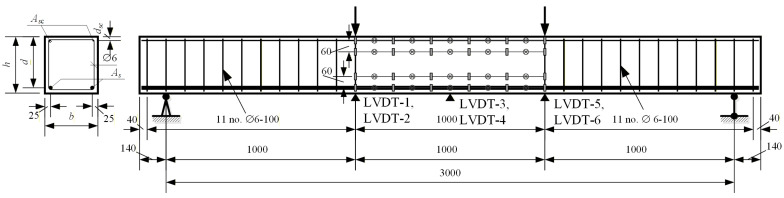
Experimental concrete beam S3-PC2 reinforced with HSS bars.

A reinforced concrete beam with a rectangular cross-section of 280 × 300 mm and a total length of 3.28 m with a clear span of 3.0 m was tested under four-point bending. The loading configuration created a 1.0 m pure bending zone between the loading points and two 1.0 m shear spans. The tension zone was reinforced with two 12 mm HSS bars, and the compression zone was reinforced with two 6 mm bars, while the shear spans contained 6 mm stirrups at a 100 mm spacing. To avoid transverse restraint effects on cracking, no stirrups were provided in the pure bending zone. The clear concrete cover to the tensile reinforcement was 25 mm. The main material and geometric parameters are given in Table 1, Table 2, Table 3 and Table 4 and Figure 2.

### 2.2. Materials

**Figure 3:**
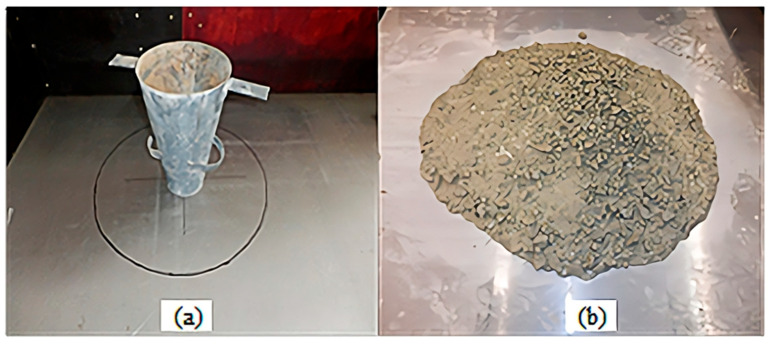
Slump flow test: (**a**) standard slump cone prior to lifting; (**b**) flow spread of concrete.

**Figure 4:**
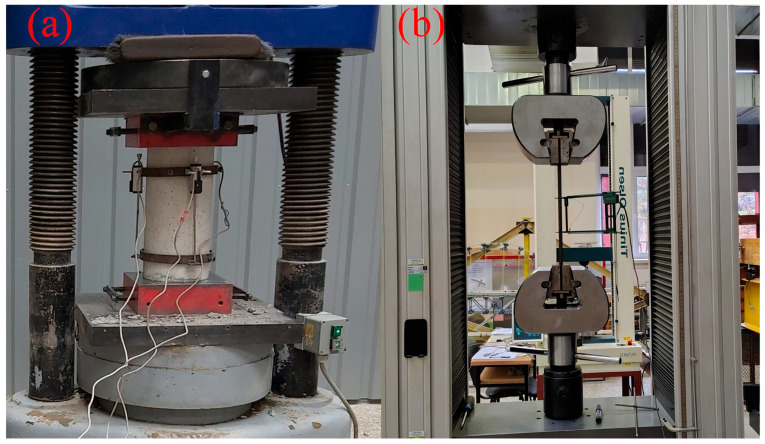
(**a**) Concrete cylinder test; (**b**) uniaxial tensile test for steel bar.

**Figure 5:**
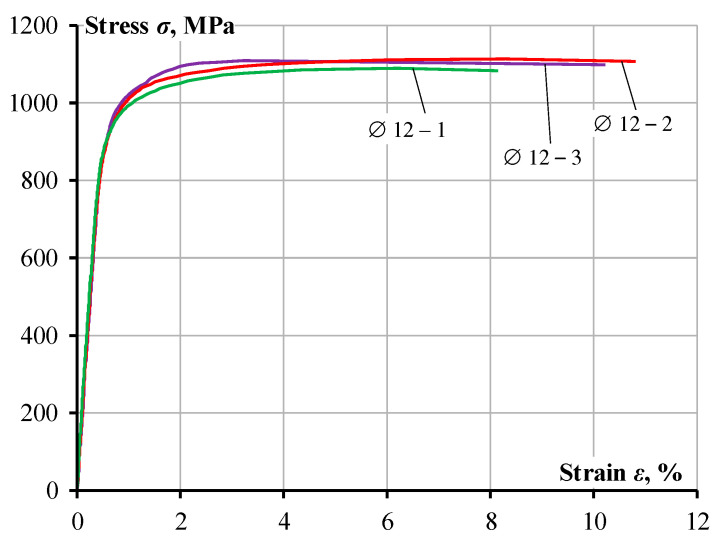
Stress–strain curve of steel bar A800.

The experimental beam was cast using ready-mixed plain concrete manufactured by UAB “Markuciai”, Vilnius, Lithuania. Table 4 presents details of the dry composition of the concrete mix used in this experimental study. The concrete mix was proportioned to achieve enough workability. Fresh concrete properties were evaluated using the slump flow test. No signs of segregation, such as irregular coarse aggregate distribution, exposed aggregate particles, or paste separation, were observed. The slump flow spread of approximately 600 mm was measured (Figure 3) and the average time to reach 500 mm spread was 20 s, which indicates satisfactory workability characteristics. The beam specimen was casted in timber frameworks and demolded 2–3 days after casting. Following demolding, the specimen was cured under ambient laboratory conditions at an average relative humidity of 65.5% (RHm) and a temperature of 20 °C until testing. The mechanical properties of the concrete were determined using standard specimen cast before and at the time of casting of the experimental beam. Three cylinder specimens (Ø150 × 300 mm) were prepared to determine the concrete compressive strength (Figure 4a) in accordance with LST EN 12390-3 Part 3 [24] and Part 13 [25], and the modulus of elasticity of concrete was calculated according to the Eurocode formulation [10]. All specimens were cured under the same conditions as the experimental beam. The average modulus of elasticity (*E_c_*) and compressive strength (*f_c_*) measured at 28 days are presented in Table 3. Uniaxial tests were conducted on three A800 steel bar specimens with a 12 mm diameter in accordance with LST EN ISO 15630-1 (Part 1) [26] to determine the modulus of elasticity (*E_s_*) and yield strength (*f_y_*). The yield strength was determined using the 0.2% proof stress offset method, where fy is defined as the stress corresponding to a permanent plastic strain of 0.2% upon unloading. All the material properties correspond to average experimental values. The stress and strain curves obtained from testing are shown in Figure 5 for the 12 mm (A800) bar. The mechanical characteristics of the steel bars are listed in Table 2, and testing of the 12 mm rebar is illustrated in Figure 4b.

### 2.3. Experimental Setup, Instrumentations, and Results

**Figure 6:**
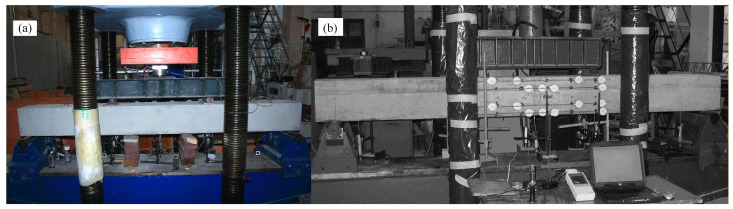
Experimental setup of the beam: (**a**) loading and (**b**) strain measurement.

An IIP-1000 hydraulic machine (VEB Werkstoffprüfmaschinen, Leipzig, Germany) with a maximum capacity of 1000 kN was employed for the four-point bending test of the simply supported experimental beam. Two-point loads were applied at one third of the clear span (L/3) of the beam through a 1250 mm long steel spreader beam, creating the pure bending zone at the mid-span of the beam. Steel bearing plates with a thickness of 10 mm were adhesively attached at each loading point to prevent local stress concentrations and premature crushing beneath the loads. The applied load was monitored using a digital load cell with a 500 kN maximum capacity. Loads were applied monotonically in increments of 2 kN under control displacement. Loading was paused at each increment for 1–2 min to record all the instrument readings, crack formation, and propagation and to mark new cracks on the beam surface. The test arrangement is shown in Figure 6.

Vertical deflections were recorded at three critical locations using linear variable differential transducers (LVDTs). A total of 6 LVDTs (AHLBORN MESS-UND REGELUNGSTECHNIK GmbH, Holzkirchen, Germany)were employed on the bottom surface of the beam and positioned along the pure bending zone, at the edges parallel to the loading direction under each loading point and mid-span. Each transducer was mounted on an independent rigid reference frame isolated from the test specimen to eliminate any influence from support settlements or rotation of the beam. ALMEMO T50 (AHLBORN MESS-UND REGELUNGSTECHNIK GmbH, Holzkirchen, Germany)was employed to record the displacements with an accuracy of ±0.15%, and for signal processing, ALMEMO 2590-9 (AHLBORN MESS-UND REGELUNGSTECHNIK GmbH, Holzkirchen, Germany) was used to capture the LVDTs and dynamometer readings every second.

Concrete surface strains were measured using 20 mechanical dial gauges with circular scales. Each dial gauge had a gauge length of 200 mm with an accuracy of ±0.003 mm and was positioned strategically within the pure bending region of the beam. The gauges were distributed at four vertical levels across the depth of the beam and five equally spaced longitudinal sections along the pure bending zone of the beam. This arrangement provided 20 measurement points in total, with detailed strain distribution across the length and depth of the beam within the pure bending region. Strain readings were recorded at each load increment throughout the test.

Formation and propagation of cracks were monitored through systematic visual inspection at each load increment. All visible cracks were marked on the beam surface using a permanent marker, and each crack was annotated with the corresponding load level. The cracking moment *M_cr_* was identified with three independent approaches: visual detection of the first visible crack; sudden changes in LVDT deflection readings, indicating stiffness reduction; and discontinuity in strain gauge measurements. The ultimate moment capacity *M_u_* was determined at the maximum applied load immediately before failure. Digital photographs were taken at various key load increments to accurately capture the crack patterns and failure modes. The moment curvature relationships were experimentally derived from measured strain distribution, and the load deflection curves were constructed by plotting applied load against mid span deflection.

**Figure 7:**
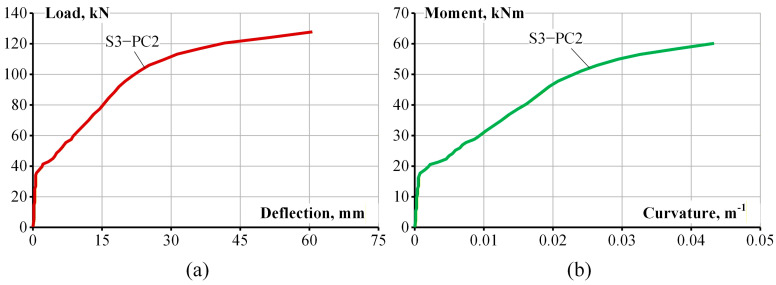
(**a**) Load–deflection and (**b**) moment—curvature graphs for experimental RC beam S3-PC2.

**Figure 8:**
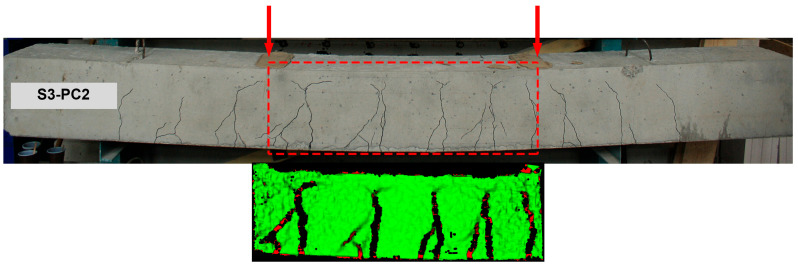
Crack pattern of experimental beam after testing.

The load–deflection diagram of RC beam S3-PC2 is presented in Figure 7a. Figure 7b shows the experimental moment–curvature diagram which will be used to inversely extract the resultant internal force of tensile concrete. Figure 8 shows the crack pattern of S3-PC2 after the test. It also displays a crack pattern of the pure bending zone obtained using the digital image correlation technique.

## 3. Collection of Test Data for Validation

In this study, an extended test dataset of 63 concrete beams reinforced with high-strength steel bars was collected from the literature. The selection criteria were based on the loading criteria and geometrical parameters of the specimens. All the beams were subjected to four-point bending, with the moment arm (*a*) being approximately one third of the clear span (*l*_0_), and the span-to-depth ratio being kept greater than 6 (*L*/*h* ≥ 6) to ensure the dominance of bending over shear behavior. Table 5 presents the main parameters of the reinforced concrete beams, such as span length (*l*_0_), section height (*h*), section width (*b*), effective depth (*d*), *d*/*h* ratio, reinforcement ratio (*ρ*), and compressive 150 mm cubic strength, (*f_cu_*). Most samples had *d*/*h* ratios close to 0.9, a typical value in this context. The experimental data, except for the current test, was presented in the form of moment–deflection diagrams. The curvature, κ, needed for the inverse analysis was expressed using the formula $\delta =s\kappa l_{0}^{2}$, where delta is the experimental deflection, *s* is the loading configuration factor, and *l*_0_ is the span of the beam. The current study (beam S3-PC2) shows how test curvature can be obtained from recordings of strain gauge continuously located at several horizontal levels along the pure bending zone.

## 4. Inverse Calculation of Tension Stiffening Force Based on Moment–Curvature Test Results

**Figure 9:**
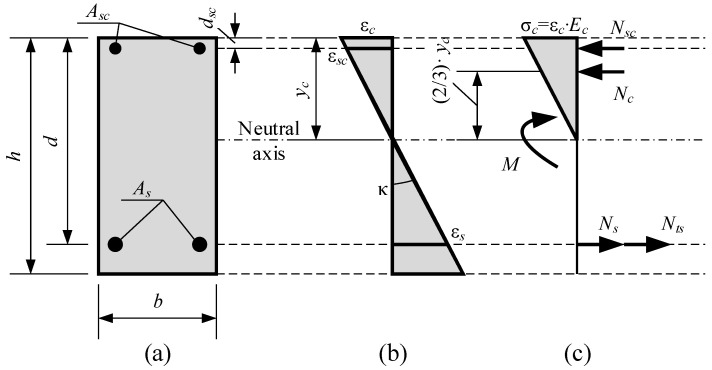
Beam with compression and tension steel subjected to bending: (**a**) cross-section; (**b**) strain compatibility; and (**c**) stresses, internal forces, and external bending moment.

Although concrete can withstand tensile stress across cracks by transferring forces from the tensile reinforcement through bonding, its tensile capacity is typically disregarded when determining the strength of a reinforced concrete beam or slab. Concrete exhibits a phenomenon called tension stiffening, which impacts the stiffness of the member once it cracks, affecting the deflection of the members [14,17]. Tension stiffening is a significant factor in determining the stiffness of the members when accurately calculating deflection and crack width at serviceability limit conditions. To quantify tension stiffening, an inverse technique was introduced by the authors and other researchers [13,18,19,21,22]. The tension stiffening inverse modeling approach described in the these references was employed in the current study and is illustrated in Figure 9. The analysis adopts the plane section hypothesis which assumes a linear strain distribution through the member’s depth (Figure 9b). Concrete behavior in the compression zone is characterized by elastic material properties. Figure 9c shows that the internal force system consists of four components: the resultant compressive concrete (*N_c_*), the compression reinforcement force (*N_sc_*), the tension reinforcement force (*N_s_*), and the tensile concrete contribution (*N_ts_*). The *N_ts_* component accounts for the combined effects of tension stiffening and tension softening of the cracked concrete. Following the approach of [17], the tensile concrete force is positioned at the centroid of the tensile reinforcement layer. The fundamental concept underlying the inverse technique involves calculation of the tensile concrete force from experimental moment curvature response. The governing relationship for this inverse analysis is expressed as follows:

(7) $$\kappa \frac{E_{c}y_{c}^{2}b}{2}\left(d-\frac{y_{c}}{3}\right)+\kappa E_{sc}\left(d-d_{sc}\right)\left(y_{c}-d_{sc}\right)A_{sc}-M=0$$

Regarding the internal force of tensile concrete *N_ts_*, Equation (7) is transformed into Equation (8) [31]:

(8) $$N_{ts}=\kappa \left[\frac{E_{c}y_{c}^{2}b}{2}+E_{sc}\left(y_{c}-d_{sc}\right)A_{s2}-E_{sc}\left(d-d_{sc}\right)A_{sc}\right]$$

where *E_c_* and *E_sc_* are the Young’s modulus of concrete and compression steel, respectively; *y_c_* is the mean compressive depth of the concrete section; *d* and *d_sc_* are the effective depth for tensile and compressive reinforcement, respectively; *M* is the bending moment; and *κ* is the experimental curvature of the reinforced concrete bending specimen.

**Figure 10:**
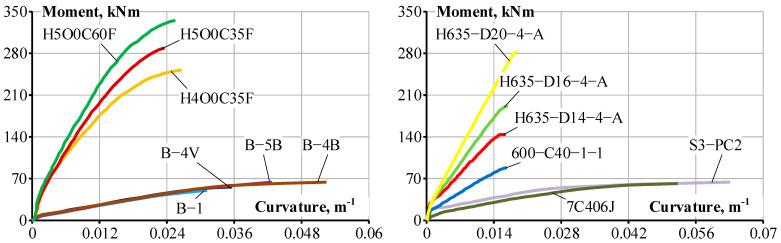
Moment–curvature diagrams of selected RC beams with HSS bars.

**Figure 11:**
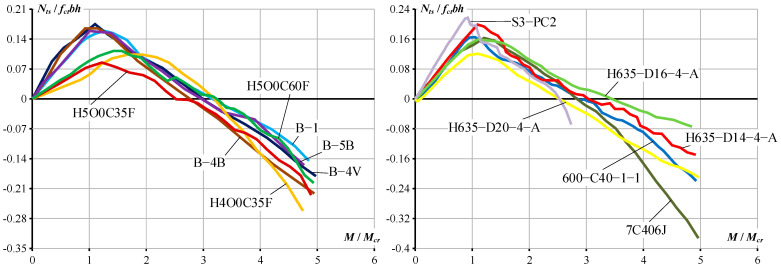
Normalized tension stiffening force versus *M*/*M_cr_* of selected RC beams with HSS bars.

The test data collected from 64 beams, including beam S3-PC2, is presented in detail in Table 6. The beams represent different experimental programs with ranging section height, cover, steel strength, bar diameter, reinforcement ratio, and concrete grade. The reinforcement yield strength (or proof yield strength) ranged from 645 to 944 MPa. The inverse analysis employed beam S3-PC2 and 12 other beams randomly selected from Table 6. Figure 10 presents the test moment–curvature diagrams, whereas Figure 11 shows the resultant tension stiffening force, *N_ts_*, versus the normalized bending moment. The bending moment is normalized relative to the cracking moment, and the resulting tension stiffening force is presented as *N_ts_*/*f_ct_bh*. The values of *f_ct_* and *E_c_* needed for the analysis are computed using Eurocode 2. The tension stiffening force has an ascending branch followed by a descending branch. The condition of *N_ts_* = 0 represents the deformation behavior of a fully cracked RC section with neglected tension stiffening. For most of the beams shown in Figure 11, the resulting tension stiffening force reaches zero (*N_ts_* = 0) at a bending moment close to 3*M_cr_*. For beam S3-PC2, investigated in the current test program, a rather low value of *β*_0_ ≈ 2.5 was obtained. Table 6 shows that this beam has the highest steel proof yield strength (*f_p_*_0.2_ = 944 MPa) and the highest concrete compressive strength (*f_c_* = 64.5 MPa) among all the test beams. The reasons why tension stiffening could be reduced in the RC members with high-strength reinforcement and high-strength concrete are as follows:

(1) High-strength concrete has increased brittleness compared to normal-strength concrete. In the latter, microcracking occurs more gradually, whereas the crack propagation in high-strength concrete is abrupt, causing a more sudden degradation in the concrete’s ability to resist tensile stresses.
(2) The aggregate interlock effect is more pronounced in normal-strength concrete with relatively low-strength cement paste and aggregates with higher strength. When a crack forms, such concrete creates a rough surface, with the crack crossing the cement paste and going around the aggregate, which transmits tensile stresses from one side of the crack to another. An effect called aggregate interlock contributes to tension stiffening. However, high-strength concrete, which includes high-strength cement paste, has a smoother crack surface, with the crack crossing both the cement paste and the aggregate. This has a negative effect on tension stiffening.
(3) The edging action of the steel bar ribs creates higher local stresses, resulting in localized crushing of the concrete at the ribs and longitudinal splitting cracks. These effects reduce bonding. As high-strength concrete has a higher modulus of elasticity, the local stresses at the rib are larger, and thus, the bond and tension stiffening degradation effects are more expressed than in normal-strength concrete.
(4) High-strength concrete generally undergoes high autogenous shrinkage that develops a pretension in the concrete, which reduces the concrete tensile capacity to resist stresses after the external load is applied.
(5) High-strength steel bars are designed with a different rib pattern to carry much higher levels of stress, which may cause larger internal (Goto) crack widths compared to standard bars.

The above effects in combination can reduce tension stiffening and the overall stiffness of the RC element.

Figure 11 demonstrates that factor *β*_0_ can be taken as 3 for high-strength steel rebars (*f_y_* > 600 MPa) as originally proposed [13] for normal-strength steel (*f_y_* = 500 MPa) rebars. However, it is important to emphasize that the current study predicted *M_cr_* according to Eurocode 2, whereas the original study [13] predicted *M_cr_* using ACI 318-14 based on the rupture modulus. This led to reduced M*_cr_*, tension stiffening, and increased curvature, as predicted by Kaklauskas and Sokolov’s model [13].

## 5. Deflection Prediction Using Different Methods

This section provides some illustrations of the moment–curvature behavior predicted using different methods, including Eurocode 2 [10], ACI 318-14 [23], ACI 318-19 [9], Bischoff’s model [8], and Kaklauskas and Sokolov’s model [13]. These models have been briefly described in the Introduction. For ACI 318-14, ACI 318-19, and Bischoff’s model, the curvature is calculated as *κ* = *M*/*E_c_ I_e_*, where the effective moment of inertia *I_e_* is determined using Equation (1) for ACI 318-14 and Equation (2) for ACI 318-19 and Bischoff’s model [8], respectively. The cracking moment *M_cr_* is calculated based on respective code provisions. The Kaklauskas and Sokolov model [13] uses the approach given in Equation (3), with the cracking moment *M_cr_* calculated according to Eurocode 2.

**Figure 12:**
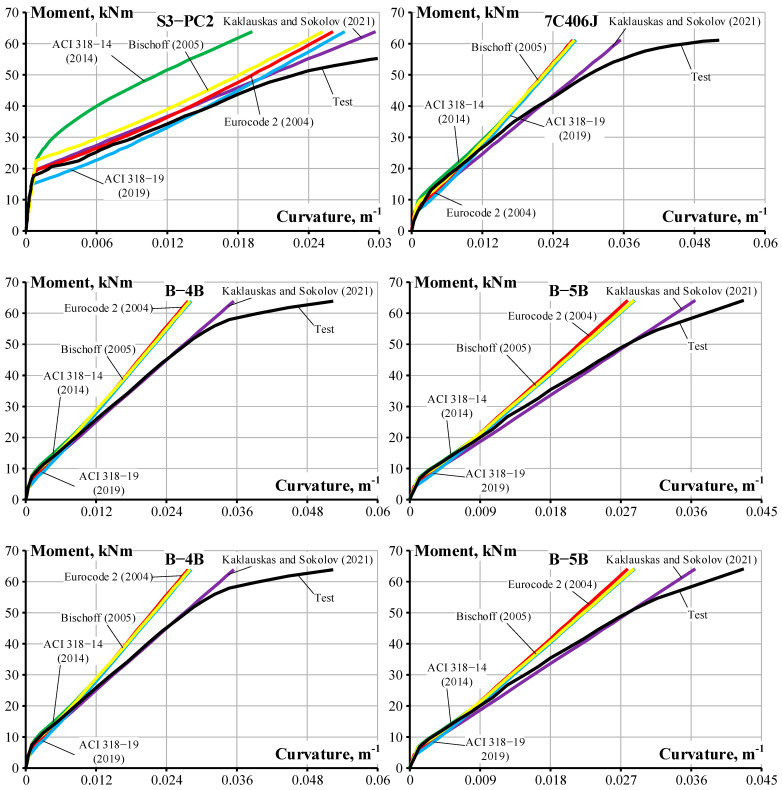

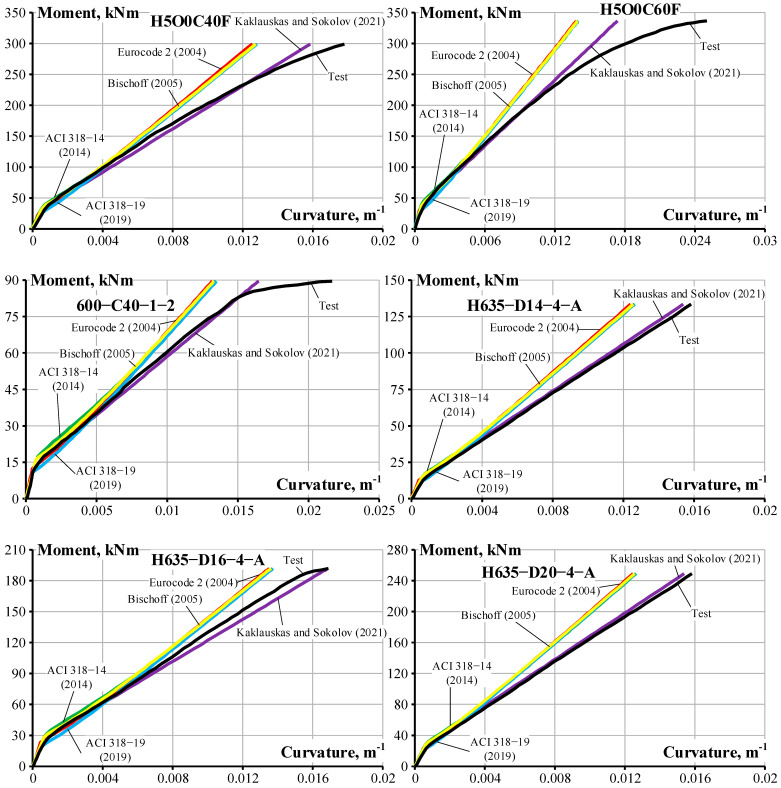
The predicted curvatures of selected test RC beams [8,9,10,13,23].

The moment–curvature diagrams for the 12 test RC beams selected from Table 6 are presented in Figure 12. The results show that the design code methods and Bischoff’s model [8] tend to underestimate curvature. The deviation starts at the bending moment around 3*M_cr_*, and the difference increases as the bending moment increases. The Kaklauskas and Sokolov model gives more accurate curvature predictions than the design code and Bischoff’s method regardless of section shape, reinforcement ratio, and concrete grade.

## 6. Statistical Analysis and Results

The earlier mentioned curvature models (Eurocode 2 [10], ACI 318-14 [23], ACI 318-19 [9], Bischoff’s model [8], and Kaklauskas and Sokolov’s model [13]) were employed in the statistical analysis to assess their predictive capability. All five models were evaluated against experimental data from 64 reinforced concrete beams tested under four-point bending schemes. The predictions are given in terms of the normalized curvature:

(9) $$\bar{\kappa }=\frac{\kappa_{\mathrm{th}}}{\kappa_{\exp}}$$

where *κ_th_* is the calculated curvature; *κ_exp_* is the experimental curvature.

Recognizing that the curvature of reinforced concrete bending members is significantly influenced by the level of stress in the steel reinforcement, the calculations were performed for multiple stress level intervals. The normalized curvature is a random variable. Therefore, normalized results can be evaluated using statistical methods. In addition, we intended to split the test data into subgroups based on different reinforcement ratios to perform statistical analysis. However, the collected test data (see Table 6) misrepresented the data of lightly reinforced members (reinforcement ratio below 0.5%); therefore, the data was considered as a single group.

At each considered stress level, the mean (*m*), standard deviation (*SD*), and coefficient of variation (*COV*) were computed for every normalized stress level using Equations (10)–(12). Statistics for each curvature calculation method are included in Table 7 and Table 8.

(10) $$m_{f}=\frac{1}{n}\sum_{i=1}^{n}\;f_{i}$$

(11) $${SD}_{f}=\sqrt{\frac{1}{n-1}\sum_{i=1}^{n}\;{\left(f_{i}-m_{f}\right)}^{2}}$$

(12) $$COV=\frac{{SD}_{f}}{m_{f}}\;$$

Confidence intervals for a normal distribution can also be expressed using the sample mean, *m*, and standard deviation, *SD*. The expected 1 − *α* confidence interval can be obtained using Equation (13):

(13) $$\left[m_{f}-t_{1-\frac{\alpha }{2}}\left(n-1\right)\frac{{SD}_{f}}{\sqrt{n}};m_{f}+t_{1-\frac{\alpha }{2}}\left(n-1\right)\frac{{SD}_{f}}{\sqrt{n}}\right]$$

**Figure 13:**
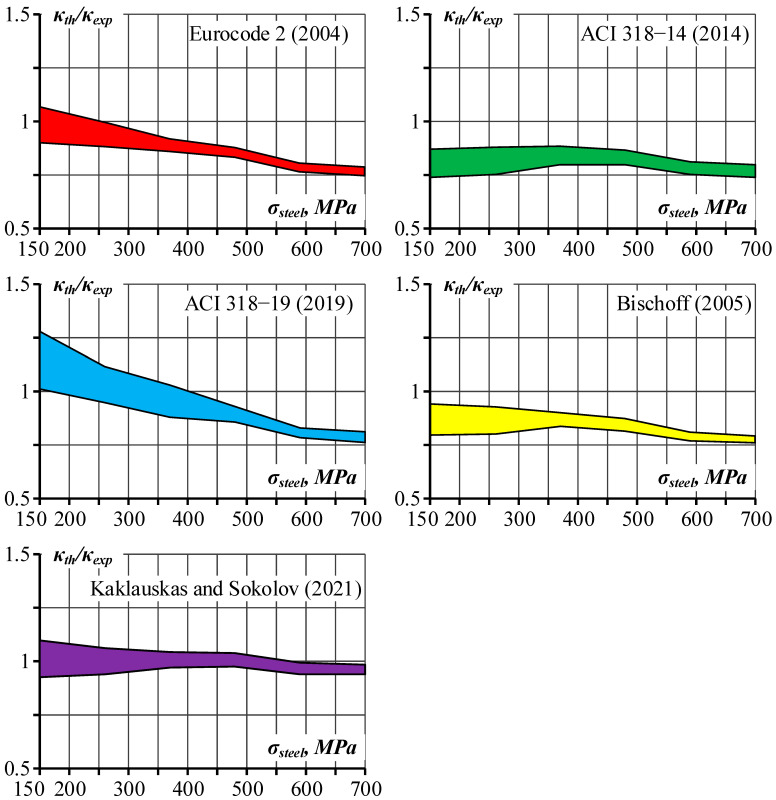
The 95% confidence interval for the normalized curvature [8,9,10,13,23].

A graphical representation of the analysis results for the 95% confidence interval for normalized curvature is given in Figure 13, in which the predicted scatter is represented by the width of the confidence interval.

Comparative statistical analysis of the curvature prediction approaches shows significant quantitative variations across stress intensity levels. As shown in Table 7, the overall performance of all specimens demonstrates substantial differences across the models. The mean normalized curvature determined using the Kaklauskas and Sokolov model is 0.993 compared to 0.875 for Eurocode 2, 0.812 for ACI 318-14, 0.946 for ACI 318-19, and 0.839 for Bischoff‘s model. The respective coefficients of variation for these models are 17.8, 20.6, 21.1, 24.9, and 19.7%.

Table 8 shows that the mean normalized curvature predicted by the Kaklauskas and Sokolov model ranges from 0.961 to 1.011 across all stress levels. The respective intervals for other models are 0.985 to 0.765 for Eurocode 2, 0.807 to 0.770 for ACI 318-14, 1.145 to 0.786 for ACI 318-19, and 0.868 to 0.776 for Bischoff‘s model. At 700 MPa steel stress, the Kaklauskas and Sokolov model on average underestimates curvature by 3.9%, while the other models’ underestimation rates range between 21.4% and 23.5%.

It should be noted that the number of beams included in the statistical evaluation decreases from 64 to 39 at the 700 MPa stress level. This reduction is attributed to two factors. First, the experimental database comprises beams collected from various studies in the literature with different design parameters, including span length, reinforcement ratio, and concrete strength, and not all beams were tested under loading conditions sufficient to produce reinforcement stresses as high as 700 MPa. Second, beams reinforced with steel with a yield strength below 700 MPa were excluded from the evaluation at this stress level, as their reinforcement could not reach this threshold. The reduction therefore reflects the natural distribution of the dataset and does not represent any selective exclusion of data.

In the current study, the Kaklauskas and Sokolov model could accurately predict deflections of the RC beam with HSS bars. Based on the normalized mean curvature, $\bar{\kappa }$, shown in Figure 13 and Table 8, values close to 1 were achieved at most stress levels of steel reinforcement. Similarly, accurate results were achieved in an earlier study [13] investigating RC beams with conventional reinforcement. The difference is that the current study assumed the material characteristics of concrete tensile strength and modulus of elasticity from Eurocode 2, whereas the previous study was based on ACI 318-19. Based on the test data in Table 6, the difference in the concrete tensile strength and *M_cr_*, as well as the tension stiffening factor, *β*_0_, defined by ACI 318-19, is on average 25% higher compared to the respective parameters predicted by Eurocode 2. Thus, it can be assumed that the tension stiffening effect in RC beams with conventional reinforcement is about 25% higher than in beams reinforced with HSS bars.

Earlier limited experimental studies suggested that beams with conventional reinforcement exhibit greater tension stiffening compared to beams with HSS bars. The current study statistically confirms this, albeit indirectly, using a large experimental dataset covering a wide range of geometrical and material characteristics. Future studies should be conducted to investigate the underlying causes of this behavior and to quantify the factors responsible for these effects.

## 7. Conclusions

This study evaluated curvature prediction models for reinforced concrete beams reinforced with HSS bars by comparing Eurocode 2, ACI 318-14, ACI 318-19, Bischoff’s model [8], and Kaklauskas and Sokolov’s model [13] against experimental data from a total of 64 beams. One beam was experimentally tested by the authors, and data on 63 specimens were collected from the published literature. The following conclusions were drawn:

1. The design codes and Bischoff’s model on average underestimate test curvatures, with the mean normalized curvature (*κ_th_*/*κ_exp_*) across several reinforcement stress levels being 0.875 for Eurocode 2, 0.812 for ACI 318-14, 0.946 for ACI 318-19, and 0.839 for Bischoff‘s model.
2. The above finding indirectly indicates that tension stiffening in RC beams with HSS bars is less pronounced compared to beams with standard steel reinforcement.
3. The inverse analysis that predicts the resultant internal force of tensile concrete (tension stiffening) from the experimental moment–curvature diagram demonstrated that on average, the stiffness of the fully cracked RC section is reached at a bending moment of 3*M_cr_*, assumed according to the EC2 design code.
4. Based on the above conclusion, in the case of HSS reinforcement, the Kaklauskas and Sokolov model assumes *M_cr_* based on Eurocode 2 specifications. This is in contrast to the original Kaklauskas and Sokolov model, which assumed *M_cr_* from ACI 318-14 based on the rupture modulus. The assumption of *M_cr_* according to Eurocode 2 reduces both the cracking bending moment and tension stiffening. In the statistical analysis, the mean normalized curvature was 0.993 for the Kaklauskas and Sokolov model.
5. The mean normalized curvature predicted using the Kaklauskas and Sokolov model ranges from 0.961 to 1.011 across all stress levels. The respective intervals for other models are 0.985 to 0.765 for Eurocode 2, 0.807 to 0.770 for ACI 318-14, 1.145 to 0.786 for ACI 318-19, and 0.868 to 0.776 for Bischoff‘s model.
6. At 700 MPa steel stress, the Kaklauskas and Sokolov model on average underestimates curvature by 3.9%, while the other models’ underestimation rates range between 21.4% and 23.5%.
7. The coefficient of variation was 20.6% for Eurocode 2, 21.1% for ACI 318-14, 24.9% for ACI 318-19, 19.7% for Bischoff‘s model, and 17.8% for the Kaklauskas and Sokolov model.
8. Current design codes require refinement to accurately predict the behavior of members with HSS reinforcement.

## Figures and Tables

**Table 1:** Main characteristics of experimental RC beam.

Specimen	*h*	*b*	*d*	*d_sc_*	*A_s_*	*A_sc_*	*ρ*
	mm				mm^2^		%
S3-PC2	300	280	277	23	226	56	0.3

**Table 2:** Mechanical characteristics of steel reinforcement.

Steel Rebar Diameter (mm)	Proof Yield Strength, *f*_*p*0.2_	Ultimate Strength, *f_u_*	Elastic Modulus of the Tension Rebars, *E_s_*
	MPa		
12	944	1104	194,155

**Table 3:** Mechanical characteristics of the concrete used in the experimental program.

Age (Days)	Cylinder Strength, *f_c_*	Elastic Modulus of Concrete, *E_c_*
	MPa	
28	64.5	40,180

**Table 4:** Mix proportions of concrete.

Material	kg/m^3^
Cement CEM-I 42.5 N	380
Crushed aggregate 4/16 mm	975
Sand 0/4 mm	885
Water	160
Concrete plasticizers	1.7
Concrete retarder	1.9

**Table 5:** Ranges of main characteristics for the experimental test programs analyzed.

Test Programs	Number of Beams	*h*	*b*	*d*	*d/h*	*l*_0_	*a*	*ρ*	*f_cu_*	*f_y_*
		mm			-	m		%	MPa	
Artemjev [27]	10	252–263	176–187	222–235	0.88–0.90	3.0	1.0	0.80–0.91	21.3–47.9	776–882
Zhang et al. [28]	8	450	250	414	0.92	3.0	1.0	0.88–1.10	36.3–60.2	711
Sha et al. [4]	11	450	250	408–411	0.91	4.5	1.5	0.74–1.93	45.2–60.2	635
Giduquio et al. [6]	8	460	330	400	0.87	4.0	1.6	1.15–1.27	28–42	690
Yao et al. [29]	16	350	200	311–313	0.89	2.55	0.875	0.49–0.82	37.7–67.1	645
Liu et al. [30]	10	400	250	353–360	0.88–0.90	3.6	1.2	0.68–1.41	52.7	650–671
Total:	63	252–450	176–330	222–414	0.88–0.92	2.55–4.5	0.875–1.6	0.49–1.93	21.3–67.1	635–882

**Table 6:** Main characteristics of test specimens employed in the analysis.

Test Programs	Specimen	*h*	*b*	*d*	*l*_0_	*c*	Ø	*A_s_*	*A_sc_*	*ρ*	*f_c_*	*f_y_*	*E_s_*
		mm						mm^2^		%	MPa		GPa
Current study	S3-PC2	302	300	253	3000	44	12	226	56	0.3	64.5	800	194
Artemjev [27]	B-1	256	176	230	3000	19	14	368	77	0.91	21.3	798	193
	B-2	263	187	235	3000	21	14	352	77	0.80	41.5	820.1	193
	B-3V	253	180	224	3000	22	14	349	77	0.87	31.1	776	193
	B-4V	252	180	222	3000	23	14	355	77	0.89	47.9	804.4	193
	B-3Ba	260	179	232	3000	21	14	367	77	0.88	28	837.3	193
	B-4Ba	255	179	226	3000	22	14	364	77	0.90	43	852	193
	B-5Ba	257	180	228	3000	22	14	361	77	0.88	35.9	831.9	193
	B-3B	255	179	226	3000	22	14	362	88.4	0.89	28.4	872.1	193
	B-4B	252	180	222	3000	23	14	360	88.4	0.90	46.6	881.9	193
	B-5B	252	179	224	3000	21	14	360	88.4	0.90	33.3	842.2	193
Zhang et al. [28]	H4O0C35F	450	250	414	3000	28	17	908	101	0.88	36.3	711	190
	H4O0C40F	450	250	414	3000	28	17	908	101	0.88	45.2	711	190
	H4O0C50F	450	250	414	3000	28	17	908	101	0.88	53.8	711	190
	H4O0C60F	450	250	414	3000	28	17	908	101	0.88	60.2	711	190
	H5O0C35F	450	250	414	3000	28	17	1135	101	1.1	36.3	711	190
	H5O0C40F	450	250	414	3000	28	17	1135	101	1.1	45.2	711	190
	H5O0C50F	450	250	414	3000	28	17	1135	101	1.1	53.8	711	190
	H5O0C60F	450	250	414	3000	28	17	1135	101	1.1	60.2	711	190
Sha et al. [4]	B1-18-A-C40	450	250	411	4500	30	18	763	226	0.74	45.2	635	200
	B2-25-B-C40	450	250	408	4500	30	25	982	226	0.96	45.2	635	200
	B3-22-C-C40	450	250	409	4500	30	22	1140	226	1.12	45.2	635	200
	B4-25-D-C40	450	250	408	4500	30	25	1470	226	1.45	45.2	635	200
	B5-22-A-C40	450	250	409	4500	30	22	760	226	0.74	45.2	635	200
	B6-20-B-C40	450	250	410	4500	30	20	942	226	0.92	45.2	635	200
	B7-22-D-C40	450	250	409	4500	30	22	1520	226	1.49	45.2	635	200
	B8-25-E-C40	450	250	408	4500	30	25	1960	226	1.93	45.2	635	200
	B9-22-C-C60	450	250	409	4500	30	22	1140	226	1.12	60.2	635	200
	B10-22-D-C60	450	250	409	4500	30	22	1520	226	1.49	60.2	635	200
	B11-25-D-C60	450	250	408	4500	30	25	1470	226	1.45	60.2	635	200
Giduquio et al. [6]	I-S1	460	300	400	4000	38	25.4	1520	253	1.27	28	690	200
	I-S2	460	300	400	4000	38	25.4	1520	253	1.27	28	690	200
	I-A1	460	300	400	4000	38	25.4	1520	397	1.27	28	690	200
	I-A2	460	300	400	4000	38	25.4	1520	397	1.27	28	690	200
	II-S1	460	330	400	4000	38	25.4	1520	253	1.15	35	690	200
	II-S2	460	330	400	4000	38	25.4	1520	253	1.15	35	690	200
	II-A1	460	330	400	4000	38	25.4	1520	1010	1.15	42	690	200
	II-A2	460	330	400	4000	38	25.4	1520	1010	1.15	42	690	200
Yao et al. [29]	600-C30-1-1	350	200	311	2550	30	18	509	308	0.82	37.7	645.02	200
	600-C30-1-2	350	200	311	2550	30	18	509	308	0.82	37.7	645.02	200
	600-C30-2-1	350	200	313	2550	30	14	308	308	0.49	37.7	645.02	200
	600-C30-2-2	350	200	313	2550	30	14	308	308	0.49	37.7	645.02	200
	600-C40-1-1	350	200	311	2550	30	18	509	308	0.82	42.6	645.02	200
	600-C40-1-2	350	200	311	2550	30	18	509	308	0.82	42.6	645.02	200
	600-C40-2-1	350	200	313	2550	30	14	308	308	0.49	42.6	645.02	200
	600-C40-2-2	350	200	313	2550	30	14	308	308	0.49	42.6	645.02	200
	600-C50-1-1	350	200	311	2550	30	18	509	308	0.82	53	645.02	200
	600-C50-1-2	350	200	311	2550	30	18	509	308	0.82	53	645.02	200
	600-C50-2-1	350	200	313	2550	30	14	308	308	0.49	53	645.02	200
	600-C50-2-2	350	200	313	2550	30	14	308	308	0.49	53	645.02	200
	600-C60-1-1	350	200	311	2550	30	18	509	308	0.82	67.1	645.02	200
	600-C60-1-2	350	200	311	2550	30	18	509	308	0.82	67.1	645.02	200
	600-C60-2-1	350	200	313	2550	30	14	308	308	0.49	67.1	645.02	200
	600-C60-2-2	350	200	313	2550	30	14	308	308	0.49	67.1	645.02	200
Liu et al. [30]	H635-D144-A	400	250	360	3600	25	14	616	226	0.68	52.7	684.3	203.5
	H635-D144-B	400	250	360	3600	25	14	616	226	0.68	52.7	684.3	203.5
	H635-D164-A	400	250	359	3600	25	16	804	226	0.90	52.7	670.7	201.7
	H635-D164-B	400	250	359	3600	25	16	804	226	0.90	52.7	670.7	201.7
	H635-D166-A	400	250	359	3600	25	16	1210	226	1.34	52.7	670.7	201.7
	H635-D166-B	400	250	359	3600	25	16	1210	226	1.34	52.7	670.7	201.7
	H635-D204-A	400	250	357	3600	25	20	1260	226	1.41	52.7	650	205.6
	H635-D204-B	400	250	357	3600	25	20	1260	226	1.41	52.7	650	205.6
	H635-D282-A	400	250	353	3600	25	28	1230	226	1.4	52.7	665	204.3
	H635-D282-B	400	250	353	3600	25	28	1230	226	1.4	52.7	665	204.3

**Table 7:** Basic statistics (mean, standard deviation, and coefficient of variation) for curvature prediction of all reinforced concrete beams.

Eurocode 2 [10]			ACI 318-14 [23]			ACI 318-19 [9]			Bischoff [8]			Kaklauskas and Sokolov [13]		
Mean	SD	COV	Mean	SD	COV	Mean	SD	COV	Mean	SD	COV	Mean	SD	COV
0.875	0.177	0.206	0.812	0.171	0.211	0.946	0.235	0.249	0.839	0.166	0.197	0.993	0.177	0.178

**Table 8:** Basic statistics (mean and coefficient of variation) for curvature prediction of all reinforced concrete beams at each stress level in steel.

Stress Level	No.	Eurocode 2 [10]		ACI 318-14 [23]		ACI 318-19 [9]		Bischoff [8]		Kaklauskas and Sokolov [13]	
		Mean	COV	Mean	COV	Mean	COV	Mean	COV	Mean	COV
150	64	0.985	0.301	0.807	0.293	1.145	0.414	0.868	0.299	1.011	0.295
200	64	0.937	0.210	0.818	0.280	1.031	0.283	0.865	0.255	0.999	0.216
300	64	0.886	0.118	0.843	0.184	0.953	0.282	0.870	0.130	1.006	0.126
400	64	0.853	0.097	0.833	0.152	0.894	0.145	0.844	0.131	1.005	0.109
600	64	0.784	0.098	0.783	0.132	0.806	0.108	0.789	0.096	0.965	0.101
700	39	0.765	0.075	0.770	0.110	0.786	0.084	0.776	0.064	0.961	0.067

## Data Availability

The original contributions presented in this study are included in the article. Further inquiries can be directed to the corresponding author.
